# Droplet Digital PCR: A Powerful Tool for Accurate Quantification of Hepatitis D Virus RNA Levels and Verification of Detection Limits

**DOI:** 10.1111/jvh.70036

**Published:** 2025-05-15

**Authors:** L. Sandmann, L. Windzio, B. Bremer, S. Falak, J. Beheim‐Schwarzbach, A. Kummrow, M. Cornberg, H. Wedemeyer, B. Maasoumy, E. Valiente

**Affiliations:** ^1^ Department of Gastroenterology, Hepatology, Infectious Diseases and Endocrinology Hannover Medical School Hannover Germany; ^2^ Excellence Cluster RESIST Excellence Initiative Hannover Medical School Hannover Germany; ^3^ D‐SOLVE Consortium, an EU Horizon Europe Funded Project (No. 101057917) Hannover Germany; ^4^ Department 8.3, Biomedical Optics Physikalisch‐Technische Bundesanstalt Berlin Germany; ^5^ German Center for Infection Research (DZIF) Hannover‐Braunschweig Germany; ^6^ Center for Individualised Infection Medicine (CiiM) Hannover Medical School/Helmholtz Center Hannover Germany

**Keywords:** HDV, HDV RNA, quantification, RT‐dPCR, RT‐qPCR, verification

## Abstract

Reliable quantification of hepatitis D virus (HDV) RNA levels is necessary for initiating and guiding antiviral treatment. The aim of this work is to develop and validate a digital PCR method for the accurate quantification of HDV RNA, including evaluation of its clinical accuracy, especially for low‐concentrated clinical samples. The reverse transcription digital PCR (RT‐dPCR) development followed the standard procedure, including primer design, determination of linearity, calculation of recovery and the intermediate precision of the RNA extraction kits, determination of the limit of detection (LOD) and quantification (LOQ), droplet size measurements, conversion factor, and uncertainty budget. The World Health Organisation (WHO)‐HDV international standard was used for RT‐dPCR development. Commutability of the new method was explored, comparing RT‐dPCR with quantification assays applied in clinical routine using clinical plasma samples covering a range of HDV RNA concentrations. The conversion factor from copies/mL to IU/mL was 0.77. LOD and LOQ of the RT‐dPCR were 0.7 copies/mL (0.56 IU/mL) and 10 copies/mL (8 IU/mL), respectively. When evaluating the qualitative results of the clinical HDV samples at low concentrations, 31% of the HDV clinical samples tested negative by RT‐qPCR were tested positive by RT‐dPCR. The RT‐qPCR and RT‐dPCR quantitative data showed a good correlation with a standard deviation of ±1.12 log IU/mL. RT‐dPCR is an accurate method for HDV RNA quantification that may serve as a complement to RT‐qPCR, especially when accurate detection is essential for decision making in clinical settings.

## 1 Introduction

Chronic hepatitis D virus (HDV) infection frequently leads to liver cirrhosis and associated complications [1, 2]. HDV infection is caused by co‐infection of the hepatitis B virus (HBV) and HDV [3, 4, 5]. Although it is named an orphan disease in Central and Western Europe, up to 19 million individuals worldwide are chronically infected [6].

Current treatment options include pegylated interferon alfa (PEG‐IFNa) and/or the entry inhibitor bulevirtide, which is available in European countries. Still, numerous questions remain about optimal treatment management and duration [7, 8, 9]. Reliable detection of HDV RNA is mandatory for treatment initiation, and accurate quantification is essential to evaluate treatment response [10, 11]. Especially in the context of defining stopping criteria of the currently still undetermined duration of bulevirtide treatment, the reliable identification of truly negative samples might become of clinical relevance. Recent data suggest that HDV RNA undetectability by RT‐qPCR during bulevirtide and interferon combination treatment is a predictor of maintained treatment response after stopping treatment [12]. However, due to non‐standardised detection methods and inter‐assay variability, viral load varies by assay modality [13].

Since 2013, a WHO standard for HDV quantification has become available [14]. However, many reverse transcription quantitative PCR (RT‐qPCR) in‐house tests show poor performance and great variability [15]. Studies demonstrated that low‐level HDV viremia only detectable by a high‐sensitive assay is associated with viral relapse after PEG‐IFNa treatment [16]. Furthermore, extraction methods play an important role in the determination of viral load [17]. National and international guidelines point out the high degree of heterogeneity in sensitivity and specificity across HDV assays and recommend using CE‐certified HDV RNA detection tests [10, 11, 18]. However, so far, methods to quantify HDV viremia are not harmonised.

Digital PCR (dPCR) is a third generation PCR technology that enables absolute quantification without calibration [19, 20]. The dPCR technology employs microfluidics to partition template DNA into thousands of independent amplification reactions that are expected to contain zero or one template molecule [20]. End‐point fluorescence reactions result in a binary output of partitions that are either negative or positive for the template. Poisson statistics can be used to correct for the possibility of multiple templates being partitioned together and can accurately estimate the absolute concentration of template DNA [21, 22].

In this work, we develop, validate, and extensively characterise a one‐step reverse transcriptase digital PCR (RT‐dPCR)‐based measurement method. We perform a quantitative and qualitative comparison of RT‐dPCR and RT‐qPCR of HDV clinical samples with different levels of HDV viremia. Digital PCR allows us to assign concentration values in an absolute approach (copies/mL) and relative to the WHO standard (IU/mL).

## 2 Material and Methods

### 2.1 Primers and Probe Design

Primers and probe were designed using Primer3Plus software (https://www.primer3plus.com/) from conserved regions of 32 aligned sequences of the eight HDV genotypes relying on HDV complete genomes (NCBI Reference Sequence: NC_001653, obtained from GenBank). Sequence and position of primers and probe were Primer Forward HDVPTB_forward: 5′ TCCCTTAGCCATCCGAGTG 3′ (820–838), primer Reverse HDVPTB_reverse: 5′ CTTCTTTCCTCTTCGGGTCG 3′ (897–916), and Probe 5′ FAM‐ CTCCTTCGGATGCCCAGGTC‐BHQ1 3′ (849–868).

### 2.2 Digital PCR Development and Optimization

Droplet digital PCR was performed on the Bio‐Rad's QX200 Droplet Digital PCR system (BIO‐RAD Laboratories, Hercules, CA, USA) and the Naica System (Stilla Technologies, Villejuif, France), using the One‐Step RT‐digital droplet (dd)PCR Advanced kit for Probes (BIO‐RAD Laboratories, Hercules, CA, USA), according to manufacturer's instructions. Further information on the digital PCR development and optimisation is provided in the Supporting Information.

### 2.3 dPCR Platform Comparison

Two platforms to partition the reaction by water‐in‐oil droplets, the QX200 Droplet Digital PCR System (Bio‐Rad) and Naica System (Stilla Technologies), were used. The difference between these two platforms is that the QX200 uses manual droplet generation while the Stilla System has chips with automatically produced droplets. The composition of the reaction mixture, template panel, and software used are provided in the Supporting Information. For comparison, three vials of WHO HDV RNA material, the same primers and probe, and the same PCR conditions including the same PCR reaction volume were used in both platforms.

### 2.4 Droplet Volume Measurements

Droplet size distributions for QX200 and Stilla Technologies assays were measured by optical microscopy. Description of the method and measurements is provided in the Supporting Information.

### 2.5 Limit of Detection and Quantification

Fourteen two‐fold dilution steps starting from 1:32 were used. Twenty replicates of each dilution were measured by RT‐dPCR in four separate runs and five technical replicates. Fifty replicates were measured for each of the last two dilutions in five separate runs and ten technical replicates. Negative template control (NTC) was included in each run with at least one NTC per 8‐well. The RT‐dPCR values were used to calculate the assigned copy number of the HDV target for the dilution series. The LOD and LOQ concentration for HDV RNA in the sample material was determined as described by [23].

### 2.6 WHO‐HDV International Standard and Measurement of Uncertainty

The first WHO‐HDV international standard (PEI‐Code 7657/12, Paul‐Ehrlich Institute, Langen, Germany), which is the standard for nucleic acid amplification technique‐based assays, was used to calculate the conversion factor from copies/mL or 1/mL to IU/mL. The standard was reconstituted for the concentration of 575,000 IU/mL.

To calculate the conversion factor and uncertainty, three different WHO HDV samples were used, and three different dilutions were produced (1:16, 1:32, 1:64) (see Supporting Information).

### 2.7 Comparison of RNA Extraction Kits

The performance of two different RNA extraction kits was analysed. Each of the two extraction methods was repeated on three different days, with three different WHO samples extracted on each day always including a negative extraction control (Primerdesign, USA). All extracted RNA samples were stored at −80°C. For INSTANT virus RNA/DNA kit (INSTANT DNA/RNA kit, 847–0259200602 Roboscreen GmbH) and QIAamp Viral RNA Mini Kit (QIAamp kit, 52,904, QIAGEN, Valencia, CA, USA), the extraction method was carried out following the manufacturer's instructions. Finally, 60 μL of RNase‐free water was added to the extracted RNA and used for further amplification. Further details are described in the Supporting Information.

### 2.8 Clinical Samples

To evaluate the clinical applicability of the developed RT‐dPCR, 42 plasma samples from patients with chronic HDV infection were analysed. RT‐dPCR results were compared to the standard HDV RNA quantification assay used in the clinical routine (Manual extraction by INSTANT virus RNA/DNA kit, RoboGene HDV quantification kit 2.0 (Roboscreen GmbH, Leipzig, Germany), real‐time PCR for quantification by LightCycler 480 II (Roche Diagnostics; LOD 14 IU/mL, linear range 100‐2 × 10^9^ IU/mL)). Chronic HDV infection was defined as HBsAg and anti‐HDV positivity or detectable HDV RNA for at least six months. Samples were grouped into high viremic (HDV RNA ≥ 60,000 IU/mL; *n* = 6), medium viremic (300–59,999 IU/mL; *n* = 9) and low viremic (< 300 IU/mL; *n* = 7) samples. Additionally, samples with detectable, but not quantifiable HDV RNA (pos, nq) (*n* = 7) and undetectable HDV RNA (negative) (*n* = 13) were included. One negative control sample from an HDV‐negative patient served as a negative control. No patient received antiviral treatment against HDV at the analysis time point. Informed consent was waived due to the anonymous use of left‐over samples from clinical routine. The study was approved by the Ethics committee of Hannover Medical School (10636_BO_K_2022).

### 2.9 Statistical Analysis

Statistical analyses were performed using Origin 2019 software (OriginLab Corporation, USA). Groups were compared by one‐way ANOVA test, and a *p*‐value < 0.05 was considered statistically significant. Pearson correlation coefficient (*r*) was calculated for RT‐qPCR and RT‐dPCR correlation analyses.

## 3 Results

### 3.1 RT‐dPCR Development

**FIGURE 1:**
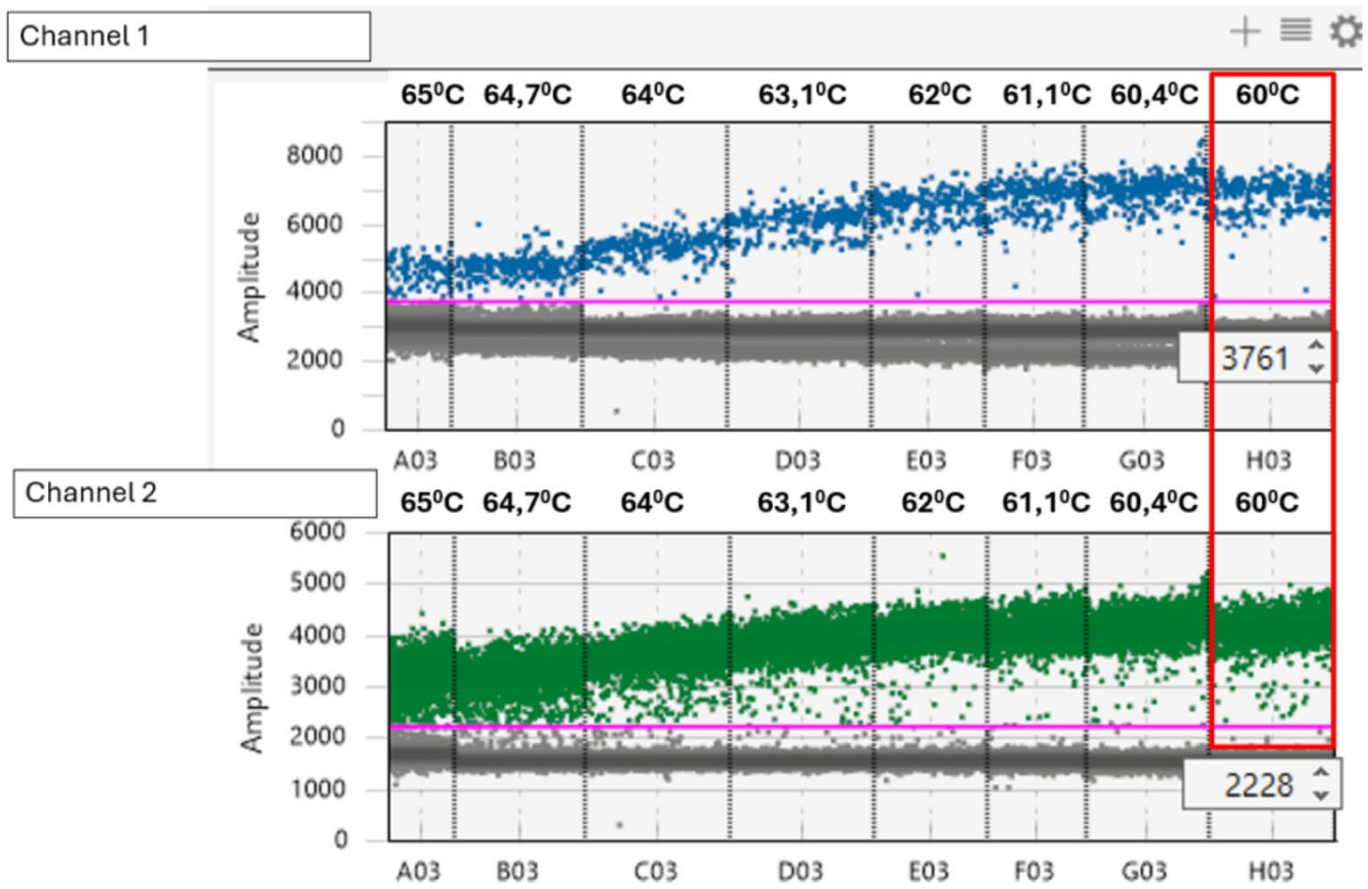
Digital PCR temperature gradient experiment showing the quantitative determination of HDV‐RNA in channel 1 (FAM) and the RNA Internal Control in channel 2 (VIC) at different temperatures from 60°C to 65°C. The pink line indicates the fluorescent cutoff (FAM: 3761, HEX: 2228). For both channels, the optimum separation of negative and positive droplets was achieved at 60°C (red rectangle).

A duplex RT‐dPCR for amplification of HDV RNA (FAM dye) and RNA extraction control target (VIC dye, Primerdesign, USA) was developed. To identify the optimal annealing temperature for the duplex RT‐dPCR, a range of temperatures (60°C–65°C) was used (Figure 1). The temperature showing the higher partitions between the positive and negative droplets and with a lower amount of rain for both dyes was selected. An annealing temperature of 60°C was selected as the optimal temperature.

The limit of detection (LOD) is defined as the WHO HDV RNA concentration, for which the probability of falsely claiming the absence of HDV RNA is 5% [24]. Digital PCR results follow a discrete distribution instead of a Gaussian distribution at low sample concentrations. LOD was calculated following the procedure described by [25]. The total number of accepted droplets was 3,290,705 (*N*_tot_), the droplet size was 0.79 nL and the dilution was 1.67, which gives *C*_LOD_ = 0.71 copies/mL (0.57 IU/mL). If we consider the LOD of just one extraction for one clinical sample (using 25 μL of sample), then LOD is 30 copies/mL (24 IU/mL).

**FIGURE 2:**
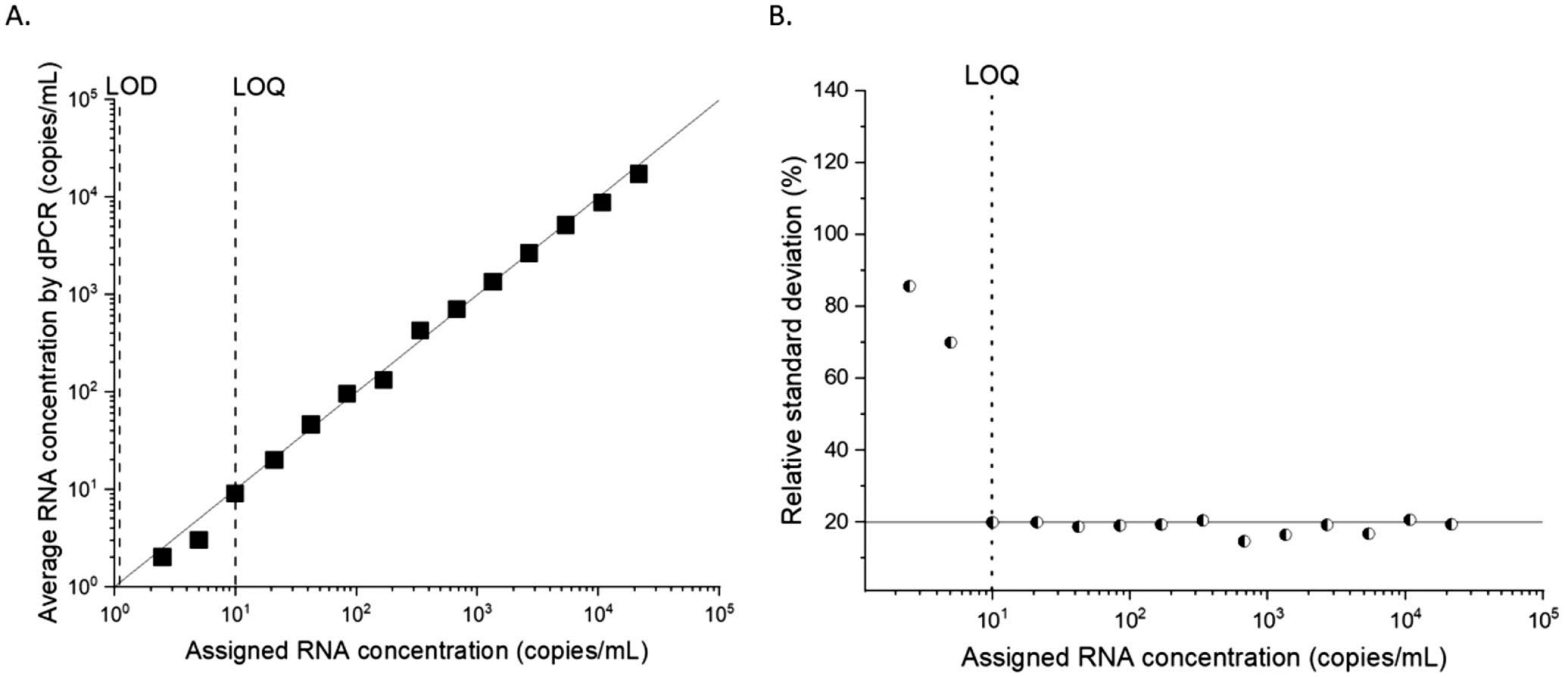
A dilution series using the WHO‐HDV standard measured by RT‐dPCR in the plasma for HDAg gene. (A) The black squares show the average concentration that closely follow expected concentrations in plasma calculated from dilution factor (solid line); (B) The relative standard deviation of replicate measurements to determine limit of quantification is shown.

The limit of quantification (LOQ) is defined as the minimum concentration for which the relative standard deviation is smaller than a predefined value *R*, e.g., *R* = 0.2 [23]. The total number of accepted droplets was 32,011,161 (*N*_tot_), the droplet size was 0.79 ± 0.01 nL, and R was set to *R* = 0.2, which gives *C*_LOQ_ = 10 copies/mL (8 IU/mL). The LOD and LOQ results, and the dilutions tested are represented in Figure 2.

### 3.2 Validation of RT‐qPCR Platforms

**FIGURE 3:**
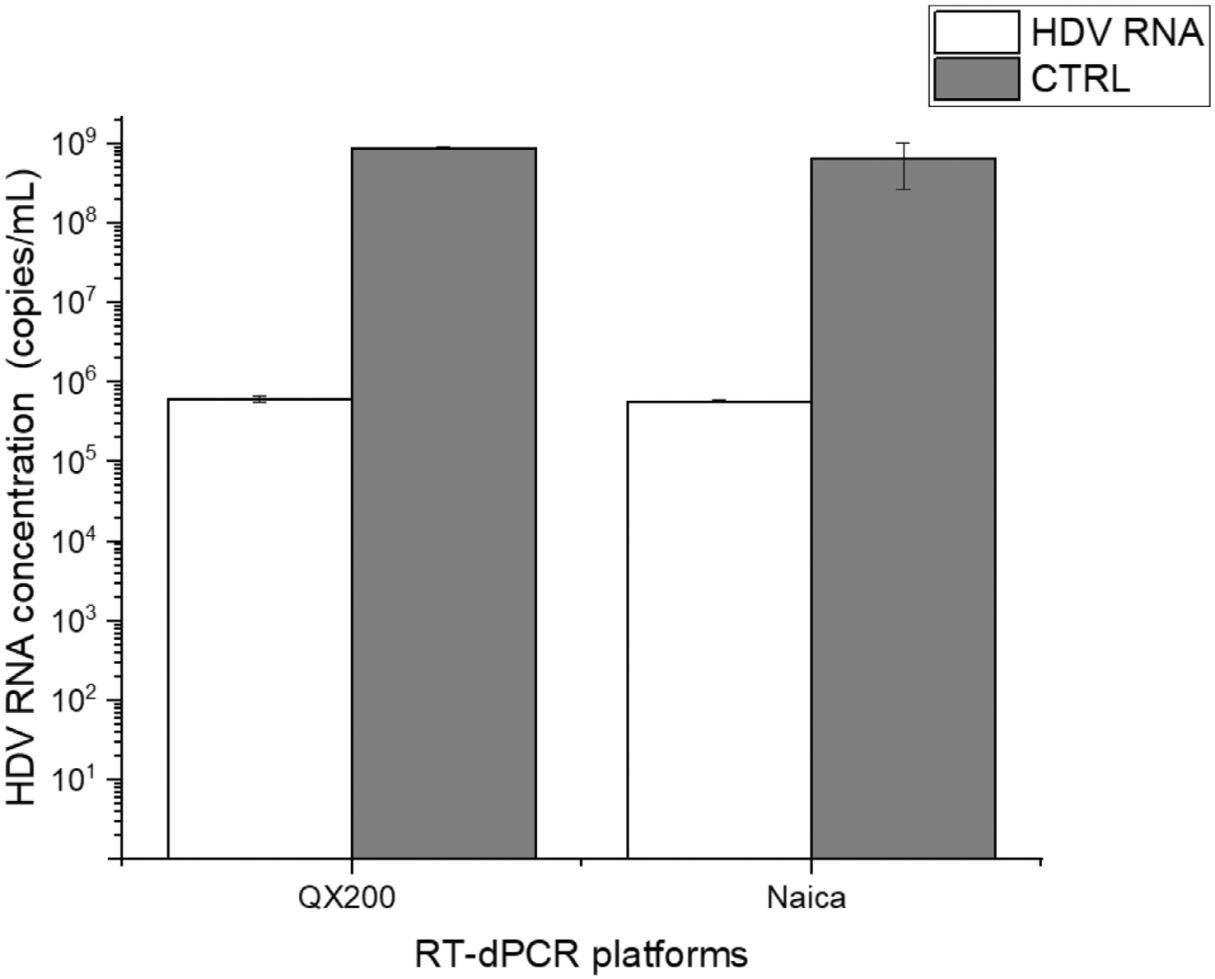
Comparison of the RT‐dPCR data from QX200 (Bio Rad) with RT‐dPCR data using the Naica Platform (Stilla Technologies). The axis *x* shows the RT‐dPCR platforms, and the y axis indicates the HDV RNA concentration in copies/mL.

To validate the QX200 platform results, similar analyses were performed using a second platform (Naica System). The droplet size measured for Naica System was 0.55 ± 0.03 nL. No significant differences in copy number concentration for HDV RNA and RNA extraction control marker were detected when comparing QX200 and Stilla technology platforms (Figure 3).

### 3.3 Evaluation of HDV RNA Extraction Kits

We evaluated the reproducibility and yield of each of the RNA extraction kits (Robogene DNA/RNA INSTANT and QIAamp Spin kits) using WHO‐HDV standard and RNA extraction control material (Primer Design, Germany). The absolute RNA concentrations of HDV RNA and RNA extraction control target were measured and the coefficient of variation (CV, %) was calculated (Table 1). The CV was lower with the INSTANT kit compared to the QIAamp kit. For clinical samples, the CV ranged from 4.6%–15% for INSTANT and 13%–26% for QIAamp kit, respectively. Due to its higher reproducibility, the INSTANT DNA/RNA kit was selected for further analyses. Considering that the INSTANT DNA/RNA kit requires a 2.5 larger sample volume (INSTANT 400 μL, QIAamp Mini Viral Kit 160 μL), the RNA quantification results were not significantly different between both kits.

**TABLE 1:** Coefficient of variation of two RNA extraction kits (INSTANT RNA/DNA and QIAamp Viral RNA Mini kit)for WHO‐HDV standard material (PEI‐Code 7657/12) and HDV clinical samples.

Materials	Coefficient of variation (%)	
	INSTANT RNA/DNA kit	QIAamp Viral RNA Mini kit
WHO PEI‐Code 7657/12	2.4–11.5	8.8–18
HDV clinical samples	4.6–15	13–26

### 3.4 Determination of Conversion Factor and Measurement of Uncertainty

The conversion factor from copies/mL to international units (IU/mL) was determined using WHO‐HDV International Standard starting from 1/16 dilution. The estimated conversion factor of the WHO HDV material from copies to international units was 0.77 (Supporting Information). The measurement uncertainty (MU) determined for WHO‐HDV measurements was 10%.

### 3.5 Comparison of RT‐qPCR and RT‐dPCR in Clinical Samples

**FIGURE 4:**
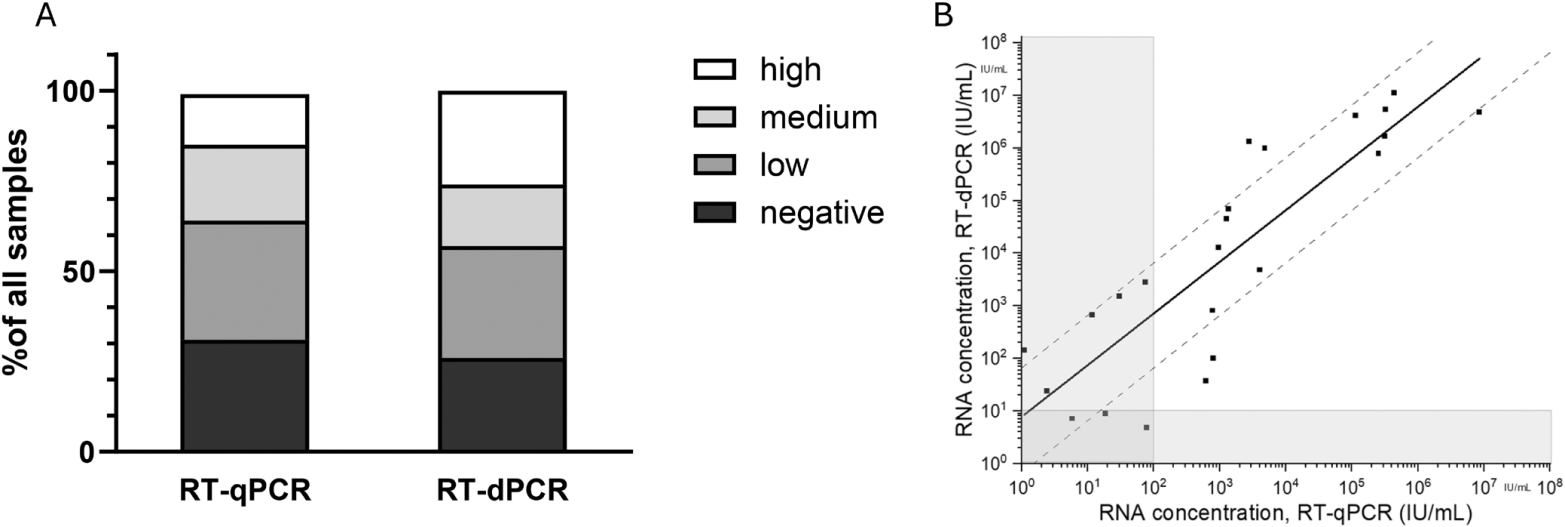
(A) Representation of the different concentration ranges (high, medium, low and negative) as a percentage of all clinical HDV samples; (B) Regression analysis of HDV‐RNA concentration values of clinical specimens obtained from HDV‐infected patients, quantified by both RT‐qPCR and RT‐dPCR. The range of ±1, 12 log deviation is indicated by dashed lines and linear regression to log values is shown as a solid line. Pearson correlation coefficient is 0.86, *p* < 0.05. Opaque area represents data below the limit of quantification.

To evaluate the diagnostic performance of the HDV RNA RT‐dPCR assay, we analysed 42 clinical samples with different levels of HDV viremia initially measured by the clinical quantification standard (Table 2). Of the 42 samples from HDV infected patients, 29 (69%) and 31 (74%) were tested positive by RT‐qPCR and RT‐dPCR, respectively. All these samples were quantifiable by RT‐dPCR, whereas only 22/29 (76%) were quantifiable by RT‐qPCR. The proportion of samples with undetectable HDV RNA was lower for RT‐dPCR compared to RT‐qPCR (26% vs. 31%, *p* = 0.80), while the proportion of high viremic samples increased (26% vs. 14%, *p* = 0.28) (Figure 4A). Four of the 13 samples (31%) that showed undetectable HDV RNA by RT‐qPCR showed detectable HDV RNA by RT‐dPCR. The correlation between RT‐qPCR and RT‐dPCR showed a Pearson correlation coefficient of 0.87 with a maximum of 1.12 log differences between RT‐qPCR and RT‐dPCR data (Figure 4B).

**TABLE 2:** List of clinical HDV samples. High concentration: > 60,000 IU/mL, medium: 300–59,999 IU/mL), low: < 100 IU/mL); pos, nq: Positive, not quantifiable.

HDV clinicals sample number	Concentration range (RT‐qPCR)	Concentration range (RT‐dPCR)
1	High	High
2	High	High
3	High	High
4	High	High
5	High	High
6	High	High
7	Medium	High
8	Medium	High
9	Medium	High
10	Medium	High
11	Medium	Medium
12	Medium	Medium
13	Medium	High
14	Medium	Low
15	Medium	Low
16	Low	Medium
17	Low	Low
18	Low	Low
19	Low	Negative
20	Low	Medium
21	Low	Medium
22	Low	Negative
23	Pos nq	Low
24	Pos nq	Medium
25	Pos, nq	Low
26	Pos, nq	Low
27	Pos, nq	Low
28	Pos, nq	Low
29	Pos, nq	Low
30	Negative	Negative
31	Negative	Medium
32	Negative	Negative
33	Negative	Negative
34	Negative	Negative
35	Negative	Negative
36	Negative	Negative
37	Negative	Negative
38	Negative	Negative
39	Negative	Negative
40	Negative	Low
41	Negative	Low
42	Negative	Low
Negative sample 1	Negative	Negative

## 4 Discussion

Reliable detection of HDV RNA is essential for both treatment initiation and evaluation of treatment response [10, 11]. However, due to non‐harmonised detection methods and inter‐assay variability, viral load varies by assay modality, making the assessments challenging [12].

In the current study, we developed and validated a RT‐dPCR for HDV RNA quantification. This included an in‐depth analysis of the analytical performance of the RT‐dPCR for HDV RNA detection and quantification, including determination of limit of detection and limit of quantification, droplet size measurements, linearity, calculation of recovery and the intermediate precision of RNA extraction kits, and calculation of measurement uncertainty. The method was validated by using a second RT‐dPCR platform and included the comparison of different HDV RNA extraction kits. Furthermore, the correlation of quantitative data of RT‐qPCR and RT‐dPCR of clinical samples with varying levels of HDV RNA was evaluated. When comparing both methods, the LOD and LOQ of RT‐dPCR were much lower than those of RT‐qPCR, indicating the high sensitivity of digital PCR. Our results indicate that the developed RT‐dPCR is a robust method for accurate absolute quantification of HDV RNA in both WHO‐HDV reference material and clinical HDV samples. However, this LOD calculation was performed according to metrology standards, which require large amounts of sample, reagents and consumables, making it challenging to apply in clinical routine. The application of RT‐dPCR for HDV RNA absolute quantification in clinical samples has been recently described [26, 27]. However, neither the described RT‐dPCR nor RNA extraction methods were evaluated. This contrasts with our study, which included the evaluation of two different dPCR platforms. Furthermore, droplet size measurement is an essential step in the development and quality control of RT‐dPCR, which was included in our approach for both tested platforms. It is important to note that the actual size of the droplets measured differed from the droplet size provided by the manufacturers (BioRad: 0.85 nL, own data: 0.79 nL; Stilla: 0.65 nL, own data: 0.55 nL). This demonstrates the need to measure droplet size volume when performing RT‐dPCR to ensure accurate measurements.

In this study, we also compared the performance of the most used RNA extraction kits in clinical settings. RNA extraction is influenced by different factors such as the capacity of the NA binding surface, purity and number of inhibitors influencing the PCR efficiency, and initial volume of the RNA extraction. The results indicate that the two RNA extraction kits evaluated show similar RNA extraction efficiency. However, the INSTANT RNA/DNA extraction kit has a lower coefficient of variation, suggesting that this kit is more reproducible than the QIAamp kit. By incorporating RT‐dPCR into RNA extraction workflows, more accurate and reproducible results might be obtained, improving the quality of RNA‐based analyses in diagnostics and clinical research.

We compared RT‐dPCR QX200 with the clinical standard RT‐qPCR by testing clinical samples of HDV‐infected patients. We observed that 31% of the samples considered negative by RT‐qPCR were detected positive by RT‐dPCR. The lower number of false‐negative tests for RT‐dPCR is particularly interesting for low viremic samples or samples with undetectable HDV RNA by RT‐qPCR. In these samples, the application of RT‐dPCR could help to identify “true negative” samples and improve the analytical accuracy, which is of clinical relevance for diagnostics and therapeutic management of patients chronically infected with HDV. Currently, HDV RNA undetectability is one of the key treatment endpoints both on‐ and off‐treatment [28]. Recent data from a clinical trial suggest that early on‐treatment HDV RNA undetectability by RT‐qPCR during bulevirtide and interferon combination treatment is a predictor of maintained treatment response after the end of treatment [29]. Furthermore, virological parameters identifying patients that can safely stop the currently life‐long bulevirtide treatment are urgently needed, as this would not only lead to cost reduction but also liberate patients from daily subcutaneous injections. The correct identification of truly undetectable HDV RNA might be an important surrogate marker for maintained off‐treatment response in this setting, and the additional analysis of selected samples by RT‐dPCR has the potential to add important information with regard to patient safety. The identification of markers predicting maintained off‐treatment response to bulevirtide treatment is currently being investigated in a multicentre, international clinical trial [30]. Decrease of false‐negative tests is important to clinical practice, and the improved diagnostic potential of dPCR found here for HDV RNA is in line with findings previously reported in a meta‐analysis of clinical trials on SARS‐CoV‐2 detection [31].

Our study has limitations. First, we only investigated two RNA manual extraction methods, which do not represent the full range of extraction kits available. However, the two selected kits are two commonly used kits and are therefore representative of the current standard in clinical practice. Second, we only used a limited number of untreated patient samples. It would be interesting to further apply the dPCR to samples from treated patients with undetectable HDV RNA or to patient samples in clinical trials evaluating new treatments. Third, RT‐dPCR consumables are still more expensive than RT‐qPCR ones, which may be less attractive to clinical laboratories, thereby limiting their applicability. Nevertheless, application to selected samples in which the identification of true negativity is important for patient management seems feasible. Additionally, samples with a high degree of mutation might be difficult to detect with the developed dPCR. However, this limitation exists in all PCR‐based quantification methods, which carry a risk of reduced primer specificity in the case of viral mutations.

In summary, we developed, validated, and well‐characterised a RT‐dPCR method to identify and accurately quantify HDV RNA levels. The performance of the RT‐dPCR shows its potential to offer a high analytical accuracy for clinical diagnostics. RT‐dPCR can also serve as a complementary method to RT‐qPCR, particularly when low viremia is suspected, and accurate detection and quantification are required for decision making in clinical settings.

## Author Contributions

**E. Valiente:** conceptualization, methodology, data analysis and writing – original draft, review and editing. **L. Sandmann:** conceptualization, methodology, data analysis and writing – review and editing. **L. Windzio:** methodology, data analysis, writing – review and editing. **B. Bremer:** methodology, writing – review and editing. **S. Falak:** methodology, writing – review and editing. **J. Beheim‐Schwarzbach:** methodology, data analysis, writing – review and editing. **A. Kummrow:** methodology, data analysis, funding acquisition, writing – review and editing. **M. Cornberg:** conceptualization, methodology, writing – review and editing. **H. Wedemeyer:** conceptualization, methodology, funding acquisition, writing – review and editing. **B. Maasoumy:** methodology, writing – review and editing.

## Conflicts of Interest

L. Sandmann reports lecture honoraria and personal fees from Falk Pharma e.V., Gilead and Roche, and travel support from AbbVie. M. Cornberg reports personal fees from Abbvie, AiCuris, Falk Foundation, Gilead, Janssen‐Cilag, GSK, MSD, Novartis, Roche. H. Wedemeyer reports grants/research support from AbbVie, Biotest, BMS, Gilead, Merck/MSD, Novartis, Roche; Personal fees from Abbott, AbbVie, Altimmune, Biotest, BMS, BTG, Dicerna, Gilead, Janssen, Merck/MSD, MYR GmbH, Novartis, Roche, Siemens. B. Maasoumy served as a speaker and/or advisory board member for AbbVIe, Fujirebio, Gilead, Luvos, MSD, Norgine, Falk Foundation, Roche, W. L. Gore & Associates and received research support from Altona, EWIMED, Fujirebio and Roche. B. Bremer, L. Windzio, S. Falak, J. Beheim‐Schwarzbach, A. Kummrow, and E. Valiente have nothing to disclose.

## Supporting information


Data S1.

## Data Availability

The data that support the findings of this study are available from the corresponding author upon reasonable request.
