# Supplementary material for: Droplet Digital PCR: A Powerful Tool for Accurate Quantification of Hepatitis D Virus RNA Levels and Verification of Detection Limits
Source: J Viral Hepat. 2025 May 15;32(6):e70036. doi: 10.1111/jvh.70036 (PMC12080313; doi:10.1111/jvh.70036)
Supplement: Supplementary file 1 — Data S1. [file JVH-32-0-s001.docx]

**Supplementary material**

[Supplementary material and methods 2](#_Toc169519903)

[Digital PCR development and optimization 2](#_Toc169519904)

[dPCR platform comparison- 3](#_Toc169519905)

[Droplet size measurement 4](#_Toc169519906)

[WHO-HDV international standard and measurement of uncertainty 5](#_Toc169519907)

[Comparison of RNA extraction kits 6](#_Toc169519908)

# Supplementary material and methods

## Digital PCR development and optimization

The reaction mixture consisted of 5 µL of One-Step RT-ddPCR Advanced kit for probes (#1864021, BioRad, USA), 2 µL Reverse Transcriptase (20U/µL), 1 µL 300mM dithiothreitol (BioRad, USA), HDV primers for HDV primers/FAM labeled probe (600 nm/250 nm), 1.4 µL of RNA control primers/VIC labeled probe mix (HB03.05.10, Primerdesign Ltd.), 4.6 µL of PCR-grade H_2_O, and 5 µL of extracted RNA in a final volume of 20 µL. The samples were analyzed in at least three technical replicates with each run including a non-template control. A total of 20 µL of each reaction mix was loaded into a sample well of a DG8 cartridge and after the addition of 70 µL droplet generation oil, droplets were generated by a QX200 droplet generator (BIO-RAD Laboratories, Hercules, CA, USA). The DG8 cartridge is composed of three rows consisting of eight wells each. The bottom wells are filled with the oil, the middle wells are loaded with the sample reaction mix and the droplets are collected in the top wells after generation. Droplets were subsequently transferred to a single 96-well plate (Eppendorf, Hamburg, Germany) and PCR amplification was performed on a C1000 Thermal Cycler (BIO-RAD Laboratories, Hercules, CA, USA). A foiled cover was used to prevent evaporation and contamination with other wells. The PCR thermal profile was set up as follows: hold 15 min at 50 °C for reverse transcription reaction, 5 min at 95 °C for enzyme activation, 40 cycles of denaturation at 95 °C for 15 s and annealing/extension at a gradient temperature (60-65°C) for 30 s (ramp rate 2 °C/s), hold 5 min at 98 °C for enzyme deactivation and final hold at 4 °C. The plate was run in a C1000 Touch Thermal Cycler according to the dPCR protocol described above. After PCR amplification, droplet fluorescence was analyzed by QX200 Droplet Reader (BIO-RAD Laboratories, Hercules, CA, USA), a two-channel detection system. Read out of droplets with positive and negative signal was performed using auto fluorescence amplitude threshold setting with the combined wells option in QuantaSoft Software (QuantaSoft version 1.7.4.0917; BioRad, USA). Wells containing less than 10000 accepted droplets were excluded from subsequent data analysis. Every dPCR run included negative template controls (NTCs) and positive template controls run in duplicate. Target DNA concentration was calculated from the fraction of positive droplets (positive end-point reactions) and number of accepted droplets using Poisson statistics [23], considering dilution factors and droplet volume. The measurement equation for calculating target RNA concentration was:

$C=D\times\lambda/{V_{d}}$

Where $C$ is HDV-*RNA* copy number concentration (copies/µL) in the dPCR premix, $D$ is the dilution factor, $\lambda$ is the average number of target RNA per droplet. The Poisson correction is

$\lambda=\text{ln}\left( 1-k/n \right)$

where $k$ is the number of positive droplets and $n$ is the total number of droplets. $V_{d}$ is the droplet volume measured in this study, and results for the final concentration were expressed as copies/mL.

## dPCR platform comparison-

For Stilla System, primers and probe concentration, volume of PCR and the PCR cycle parameters were the same as the parameter used in QX200. For Naica System (Stilla Technologies) the reaction mixture consisted of 10 µL of One-step RT PCR (2x) Quanta qScript XLT (95143-020, Quantbio), HDV primers/FAM labeled probe (600nm/250nm), 1,4 µL of RNA control primers/VIC labeled probe mix, 3,6 µL of PCR-grade H_2_O, and 5 µL of extracted RNA in a final volume of 20 µL. The same template panel for the dPCR assay comparison was prepared for the platform comparison. The samples were read by using Crystal Reader Software (Version 2.4.03), data were analyzed by Crystal Miner Software (Version 2.4.03), the droplet size was measured, and results were expressed as copies/µL of PCR reaction.

## Droplet size measurement

Freshly prepared droplets obtained with Bio-Rad droplet generator using a DG8 cartridge (Bio-Rad) were not thermally cycled prior to volume measurement as the shape of droplets change during the thermal cycling process. Therefore, dedicated negative template control samples were used for droplet sizing by transfer of generated droplets to readout chambers (ibidi µ-slide VI flat, 80621 uncoated). By contrast, the droplets generated in the Stilla Technologies Sapphire Chips are not in contact with ambient air, which allows performing thermal cycling and RT-dPCR result reading prior to volume measurement. In this assay, droplet size measurements are performed by directly transferring the Sapphire Chips to the microscope.

The microscope used for droplet size measurement (Zeiss, Axio Observer Z1) utilized a high-resolution camera (Zeiss, AxioCam 506 mono, image size 2752×2208 pixels) for digital image recording. Image scaling was determined with a microscopic ruler calibrated traceable to SI units. Measurements of droplets generated by QX200DG were measured with bright field imaging using a 10× objective (Zeiss Plan-Apochromat 10x/0,45, condenser NA 0.55). Sapphire Chips from Stilla were measured in epifluorescence using a 5× objective (Zeiss, Plan-Apochromat 5x/0.16). The fluorescence filter settings were as follows: excitation filter: band pass 450 nm to 490 nm, beam splitter and emission filter: long pass 510 nm and 515nm. Image distortion across the field of view was inspected using the traceable microscopic ruler as test object. No nonlinearities were found exceeding uncertainty limits provided by the calibration of the test object. The pixel pitch in recorded images is 0.87916 µm ± 0.00073 µm and 0.44019 µm ± 0.00067 µm for imaging with the 5× and 10× objective, respectively. Uncertainties quoted are standard uncertainties. The resulting optical resolutions available in the images for droplet sizing are 2.3 µm for fluorescence imaging with the 5× objective, and 0.7 µm for bright field imaging with the 10× objective.

A manual count was conducted in both Bio-Rad and Stilla technologies droplets using three HDV WHO vials. A total of at least 1000 droplets were counted. The average of droplets and the coefficient of variation was calculated. This measurement was considered to manually calculate the concentration of HDV RNA in the HDV WHO material. Image processing and data analysis were undertaken using ImageJ software.

## WHO-HDV international standard and measurement of uncertainty

For RT-dPCR (QX-200, Bio-Rad), the measurement uncertainty was calculated ((STDEV/mean) x100).

| WHO-HDV international standard (dilution factor) | RT-dPCR HDV-RNA (copies/mL) |
| --- | --- |
| 16 | 721628 |
| 32 | 749370 |
| 64 | 761370 |
| Mean | 744123 |
| Standard deviation | 20384 |
| Conversion factor (IU/copies) | 0.77 ± 0.02 |

Supplementary table 1. Determination of the conversion factor from copies/mL to IU/mL for RT-dPCR assays

## Comparison of RNA extraction kits

We compared the amount of RNA obtained with each extraction kit and the coefficients of variation (CVs, %) of both RNA extraction kits. To determine the intermediate precision of each extraction method, the coefficients of variation was calculated ((SD_measurement_/Mean_measurement_) *100). Intermediate precision was determined as the variability between the mean concentration (copies/mL) obtained for each of the three days of extraction (six measurements, one for each day of extraction). For the intermediate precision, the acceptability criteria were set at CV ≤20 %.
